# Considerations for Individual-Level Versus Whole-School Physical Activity Interventions: Stakeholder Perspectives

**DOI:** 10.3390/ijerph18147628

**Published:** 2021-07-18

**Authors:** Byron Tibbitts, Kathryn Willis, Tom Reid, Simon J. Sebire, Rona Campbell, Ruth R. Kipping, Rebecca Kandiyali, Russell Jago

**Affiliations:** 1 Centre for Exercise, Nutrition & Health Sciences, School for Policy Studies, University of Bristol, 8 Priory Road, Bristol BS8 1TZ, UK; kate.willis@bristol.ac.uk (K.W.); tom.reid@bristol.ac.uk (T.R.); simon.sebire@bristol.ac.uk (S.J.S.); russ.jago@bristol.ac.uk (R.J.); 2 Bristol Medical School: Population Health Sciences, University of Bristol, Bristol BS8 2PL, UK; rona.campbell@bristol.ac.uk (R.C.); ruth.kipping@bristol.ac.uk (R.R.K.); rebecca.kandiyali@bristol.ac.uk (R.K.); 3 The National Institute for Health Research Applied Research Collaboration West (NIHR ARC West), University Hospitals Bristol NHS Foundation Trust, Bristol BS1 2NT, UK

**Keywords:** physical activity, qualitative research, stakeholder perspective, whole-school approach

## Abstract

Strategies to address declining physical activity levels among children and adolescents have focused on ‘individual-level’ approaches which often fail to demonstrate impact. Recent attention has been on an alternative ‘whole-school’ approach to increasing physical activity that involves promoting physical activity throughout all aspects of the school environment. There is, however, a lack of evidence on how whole-school physical activity approaches could be implemented in the UK. This qualitative study explored perspectives of key stakeholders on potential reasons for the lack of impact of individual-level school-based interventions on children’s physical activity, and key considerations for adopting a whole-school approach. Nineteen semi-structured interviews were conducted with a range of stakeholders involved in the implementation of physical activity programmes in UK schools. Data were analysed using an inductive approach. Respondents suggested that individual-level school-based interventions to increase physical activity often failed to consult end users in the design and were typically implemented in environments unsupportive of long-term change. They subsequently outlined specific barriers and key facilitators for the adoption and implementation of whole-school approaches in UK settings and recommended a shift in research foci towards building an evidence base around educational outcomes and whole-school implementation insights.

## 1. Introduction

The physiological health benefits of being physically active are well established in children and adolescents [1,2,3,4] and the evidence linking physical activity with mental health in this age group is growing [5]. However, physical activity levels decline as children age [6] and, by adolescence, fewer than 20% of young people globally meet the WHO recommendation of an average of 60 min of moderate-to-vigorous physical activity per day [7].

Efforts to promote children’s physical activity have largely centred around school, as a pragmatic setting in which to recruit and deliver physical activity promotion to large numbers of children [8]. School-based physical activity interventions abound, but only a limited number show promise [9]. Current research has focused on individual-level (or ‘targeted’) or single behavioural context interventions that target a sub-group of pupils within a school identified as being at particular risk (e.g., girls, or a single year group) or a single intervention. However, systematic reviews have shown that most of these studies fail to elicit meaningful change in objectively measured physical activity outcomes [10]. There is a need for population-level strategies to have a marked impact on physical activity and reduce the future incidence of non-communicable diseases [11].

An alternative approach that takes more of a population perspective is to target the whole school with a range of interventions that weave physical activity opportunities throughout the school day and create an environment that is more supportive of physical activity. Some targeted interventions do act on the whole school rather than just one sub-group, such as programmes to promote active travel to school [12,13]. However, these programmes still address a single behavioural context (e.g., the journeys between home and school), and therefore, would be considered as just one component in a truly ‘whole-of-school’ approach. While this ‘whole-of-school’ approach, or comprehensive school physical activity programme (CSPAP) [14], has received recent attention, there is a need for more information about how to implement these programmes and make them maximally effective.

Since quantitative research has identified that school-based interventions are inconsistently and minimally effective at increasing physical activity but cannot provide concrete reasons for the lack of impact, we used a qualitative approach to gain the perspectives of strategic public health stakeholders to address the following three research questions in relation to the UK:

1. What are perceived reasons for the failure of traditional individual-level interventions delivered in school settings to achieve increases in children’s physical activity?
2. What are the key considerations for adopting and implementing an alternative, whole-of-school approach to increasing physical activity in UK schools?
3. How is research used by different organisations, including schools, to inform physical activity intervention development, and what further research is needed to support development and adoption of successful physical activity interventions in schools?

## 2. Materials and Methods

The methods and results are reported in accordance with the consolidated criteria for reporting qualitative research (COREQ) checklist [15].

### 2.1. Study Context and Design

This sub-study formed part of a larger UK NIHR-funded research project called PLAN-A; an individual-level, cluster-randomised controlled trial that aimed to determine whether a peer-led intervention could increase 13–14-year-old girls’ physical activity and be cost effective. The trial methods and results are published elsewhere [16,17]. Qualitative methodology was the most appropriate approach to address the three further research questions we had; thus, our current study design used qualitative methods to elicit the perspectives of strategic stakeholders (who were aware of the outcome of PLAN-A) regarding the intervention model we used, along with alternative approaches to increasing the physical activity levels of children and adolescents in all school settings. Ethical approval for the PLAN-A study and these interviews was granted by the School for Policy Studies Ethics and Research Committee at the University of Bristol (REF: SPSREC17–18.C22) and written informed consent was obtained from all participants.

### 2.2. Sampling and Participants

The participants were adults directly involved with the promotion of physical activity with young people via schools in England. This informant group was chosen because of their operational oversight of intervention implementation in schools and related settings, giving them a strategic overview of programme success or failure and an understanding of context. Participants were sampled purposively using the following criteria: the person was employed in the public or private sector in England, and their professional role directly involved the commissioning or implementation of physical activity-promoting initiatives with young people and/or working with schools to do so. Potential participants were identified in two ways. First, the research team identified local and national organisations and stakeholder groups that would include members that met the above inclusion criteria. Second, using a snowball recruitment technique [18], participants were asked to recommend other suitable candidates who could provide valuable insights and who may not have been identified by the research team.

An email with an overview of the PLAN-A study and purpose of this sub-study was sent to each potential participant. An information sheet as well as a consent form was attached. Twenty-nine people were approached, two of whom declined and suggested alternative participants, and eight did not respond. Participants therefore comprised 19 stakeholders (10 female) from the third sector, public health organisations, schools, and local government (see Table 1).

### 2.3. Data Collection

Semi-structured interviews were conducted [18]. The interviews sought to elicit stakeholders’ professional perspectives on the critical factors that influence the efficacy of physical activity interventions in school settings, potential alternatives (specifically, a whole-school approach to increasing physical activity within the school setting), and the direction that research should take to support improvements in intervention uptake and impact. The semi-structured interview guide was developed by B.T. (PLAN-A Trial Manager) and K.W. (Research Associate) in consultation with R.J. (PLAN-A Principal Investigator) and S.J.S. (PLAN-A Co-Investigator). Both B.T. (male) and K.W. (female) are experienced in conducting qualitative data collection and analysis. Focused question topics related to the three research objectives were used to increase information power [19], but questions and prompts were open-ended to prevent steering responses and to encourage detailed answers.

B.T. and K.W. conducted the interviews via phone or video-conferencing software between October 2020 and January 2021, which was during the COVID-19 pandemic. At the start of each interview, the researcher provided background information on PLAN-A, the purpose of the interviews, and disclosed their role in the project. Interviews were audio-recorded using encrypted voice recorders and ranged from 30 to 61 min in duration. The interview guide was piloted with two participants and then small refinements to the prompts were made. Field notes were made alongside each interview and used to discuss possible refinements to the interview guide after every two interviews to ensure consistency between researchers in interview style and prompts. Interviews were transcribed by an independent transcription company and then checked against the audio recording and anonymised by three research team members (B.T., K.W. and T.R. (fieldworker)).

### 2.4. Data Analysis

All coding and data management was performed using NVivo 12 Pro (QSR International (UK) Ltd, Daresbury, Cheshire WA4 4FS, UK). A general inductive approach [20] was used by the research team to inductively code and analyse the raw data. Data analysis began concurrently with data collection in order to carefully monitor and appraise inductive thematic saturation [21]. Firstly, three transcripts were read thoroughly by K.W., T.R. and B.T. Focusing on each research question separately, initial themes were identified and, after discussion, organised into a coding frame per research question. Subsequent transcripts were coded according to the coding frame by K.W. and double-coded by B.T. or T.R. to ensure agreement. If new codes emerged that fell outside of, or disagreed with the agreed coding frame, then they were revised by the three researchers and transcripts were re-read considering the new structure. After 19 interviews, the three researchers agreed that no novel insights were emerging from transcripts and additional data would not add meaningful information power, so data collection ceased. Once all transcripts had been double-coded, narrative summaries and diagrams were used to conceptualise emerging themes and explain how themes were linked, both within and between research question coding frames.

Trustworthiness was maximised by conducting checks of agreement in interpretation through double coding and verifying that different coders (K.W., B.T. and T.R.) found relevant text using the code descriptions. Additionally, the inclusion of supporting quotations from the range of participants that were interviewed further improved trustworthiness [22]. Quotes are labelled by respondent number, e.g., *RES 1*. Respondents were numbered in the order that they were recruited.

## 3. Results

Data are presented below in relation to each of the three research questions (RQs).

### 3.1. RQ1: Perceived Points of Failure of Individual-Level Interventions in Schools

Participants highlighted three key areas of failure for physical activity interventions: (1) intervention design considerations; (2) the school as the setting; and (3) the wider environment.

#### 3.1.1. Intervention Design

Several stakeholders described a ‘one-size-fits-all’ approach to intervention design that failed to consult with end users, i.e., pupils and school staff, or be flexible to their needs.

**We’ve made the mistake for years of putting on activities for young people, as opposed to understanding what the needs of the young people are, and delivering what they want.**

*RES 2*

Several stakeholders more specifically believed the identification and training of appropriate delivery staff was overlooked in many intervention designs in two ways. Firstly, respondents pointed out that school-based interventions are delivered by ‘sporty’ people that less-active pupils may struggle to relate to. Secondly, because most interventions involve external delivery agents such as sport coaches instead of training school staff and have a finite delivery period, they are not sustainable. Stakeholders also identified a failure to engage parents.

**Again, I think from the projects that we’ve done, the ones that I’m thinking of, when you could get the buy-in from the parents, the impact is huge.**

*RES 19*

#### 3.1.2. The School as the Intervention Setting

**Figure 1:**
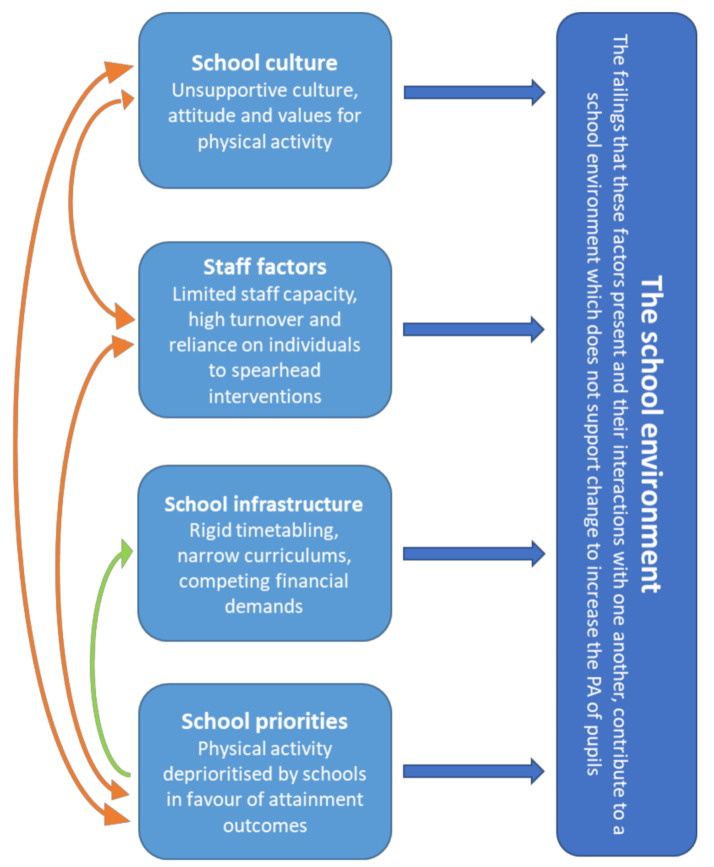
Interconnected school factors increasing the potential for intervention failure.

Some respondents proposed that the school setting may not be the best environment to conduct targeted physical activity interventions, particularly for children who are disengaged with physical activity. Multiple factors with the potential to negatively affect a pupil’s experience of physical activity in school were identified: exposure to numerous pressures and adjustments, not feeling comfortable in the school environment, and complex social interactions. These were recognised to particularly affect girls. Further to this broad viewpoint, four additional sub-themes emerged: infrastructure, school priorities, school culture and staff factors. Figure 1 depicts the interrelatedness of these factors and their contribution to intervention failure via an unsupportive school environment, as conveyed by our respondents.

Overburdened staff, a rigid school timetable or a curriculum that is geared towards competitive sport, and competing financial demands were identified as key infrastructural challenges specific to implementing interventions in schools, and therefore, achieving impact on PA.

**[…] we very often find that the curriculum is planned for the year and therefore all of the logistics and the resources associated with that, whether that be timetabling, staff expertise, equipment, all of those sorts of things are set. So, that can be a barrier for schools to make immediate change.**

*RES 8*

Stakeholders pointed out that academic subjects (e.g., Maths and English) usually take priority over PE and PA-related initiatives in schools because these are the areas that schools must report against to school boards and the Office for Standards in Education (Ofsted). This pressure to focus time, resources and energy elsewhere means individual-level physical activity interventions tend to be de-prioritised and changes made are not supported or maintained.

**I think obviously the pressures upon schools in terms of standards, progress, and achievements, obviously has a significant factor as well, when it comes to making decisions on what schools prioritise and what resource they put behind that.**

*RES 8*

Mentioned by almost all stakeholders, the school ethos and collective attitude towards PA, referred to as ‘school culture’, can support or undermine physical activity interventions. Crucially, when individual-level interventions are implemented in an unsupportive school culture, they are much less likely to succeed, nor see any positive immediate impact maintained.

**Because if your environment and your structures and your attitudes aren’t changing, I’m not sure you get any real meaningful change either.**

*RES 9*

Respondents identified Head Teachers and their senior leadership team as the key drivers of school culture. These individuals set the priorities of the school, and their behaviour and attitude towards physical activity set the tone of the school’s attitude towards interventions to increase it.

**[…] if they don’t have that top buy in right the way from the Head Teacher downwards, it can be real uphill battle for a lot of PE departments to change the way that sport is implemented.**

*RES 6*

In addition to the relatability of delivery agents (see Section 3.1.1), stakeholders identified staff capacity and turnover in schools and a reliance upon individual teachers to spearhead or support interventions as key connected issues that limited the success and sustainability of many interventions.

**I think a targeted approach could be so reliant on having an enthusiastic member of staff. […] And they do it for a bit, and then they leave, or they have to prioritise something else, and then it stops.**

*RES 15*

#### 3.1.3. The Wider Environment

Stakeholders agreed that the potential impact of individual-level interventions in schools is also subject to influence from factors beyond the school gates. Key examples of this included the availability, or lack thereof, of exit routes into community clubs for sports participation, and the quality of and access to appropriate physical activity facilities. Some stakeholders believed interventions overlook those from less affluent households who may have negative experiences of physical activity and need more support from their wider community to help overcome the additional barriers they face to being active.

**We’re finding that it’s about embedding it into the wider structures. […] It’s about that longer-term exit strategy, I suppose, from interventions.**

*RES 12*

### 3.2. RQ2: Perspectives and Points of Consideration for a Whole-School Approach to Increasing Physical Activity

Most stakeholders strongly believed that a whole-of-school approach to promoting physical activity was essential to bring about systemic changes in the culture and ethos of a school to normalise physical activity. Themes addressing this research question fell broadly within four categories: design ideas, adoption considerations, implementation and sustainability considerations. Respondents also commented on how a whole-school approach might address inequalities.

#### 3.2.1. Intervention Design

Stakeholders described many ideas for the design of a whole-of-school approach and how it could be implemented within schools (Table S1). These are organised in to three main categories: (a) broader whole-school changes (to curriculum and strategy), (b) rebranding physical activity and (c) the strategic use of role models. The table also includes stakeholders’ opinions on the groups to be consulted with in the design phase of any such intervention. It was perceived that without this consultation, the initiative would fail to provide autonomy to, and meet the needs of, pupils and the school; both of which were suggested to play vital roles in the failure of targeted interventions.

#### 3.2.2. Adoption Considerations

To encourage uptake of a whole-school approach, stakeholders felt that it was essential to have buy-in and passion for change at all levels throughout the school, but importantly from high-level, senior staff. Three strategies were discussed.

The first was to demonstrate that intervention objectives align with key measures of attainment for the school, and frame these and the whole-of-school approach in the context of enhancing health and wellbeing, as opposed to ‘delivering PE’.

**[…] if you’re going to have a whole-school approach, it has to relate to those wider measures for schools to really buy into it, certainly at senior level.**

*RES 8*

The second was to obtain high-level support from outside the school. If national or local government (e.g., Department of Health), governing bodies and key leaders in the education sector endorse the approach, schools are likely to consider adoption more seriously. Some stakeholders suggested that the objectives of a whole-of-school approach should help form Ofsted criteria.

**I think there is much for us to do at a more strategic level in terms of influencing the key decision makers who can support schools to take that more broad approach.**

*RES 6*

The final strategy was to provide evidence of the potential impact and benefits to pupils and schools.

**[…] if you can demonstrate through a case study or through piloting it somewhere else, the effect it actually has, you know, people will much more buy into that than they will if you just said, ‘Oh, we think physical activity is really important,’ […]**

*RES 5*

#### 3.2.3. Intervention Implementation

There were four common themes that stakeholders thought underpinned successful implementation.

1. *Culture change:* The values and attitudes of the school and all staff need to change in a way that will be supportive of physical activity, and therefore, of the implementation of such an approach. Table S2 presents stakeholder perceptions of how change can be achieved through creating objectives for the school as a whole but still focussing on the staff and pupils as individuals.

**[…] in order to truly improve the experience of PE for young people it needs to be this culture change that comes from the top in schools. Senior leadership engagement and endorsement is key […].**

*RES 12*

2. *Awareness and acknowledgement of key challenges:* Intervention designers and deliverers should strive to understand the key challenges that individual schools may face in implementing a whole-of-school approach to plan potential solutions or mitigate such barriers. Stakeholders identified a range of barriers, including increased pressures on staff (particularly those who are not PE teachers) to facilitate learning that promotes physical activity, particularly if they are unskilled or not confident to do so. Funding challenges and a rigid curriculum were also highlighted.

**I think staff time and teacher release is probably one of the biggest barriers. Very often, it is a challenge for teachers to come out and attend training, and quite often schools won’t be allowed to attend training because there is nothing to [cover] supply costs.**

*RES 8*

3. *Building positive relationships:* For such an approach to work effectively, respondents stressed that good working relationships between all stakeholders (school staff, external support for programme implementation, and the wider community) are essential to facilitate knowledge and value sharing.

**[…] one of the key things we do say [to schools] is make sure you have enough time to lead and coordinate something like this. The time is important to build relationships. We’re finding that building positive relationships […] between the schools is so important. Also, between staff who are working in the space, sharing learning.**

*RES 12*

4. *Providing a simple and adaptable offer:* The whole-of-school approach needs to be feasible for the school to deliver, being as efficient as possible with workload and time commitment. The more tailored the intervention is to school and pupils needs, the more likely staff will buy in and contribute, further aiding culture change throughout the school.

**[…] making sure we can be as flexible as possible for schools, to enable them to take an approach that best suits their circumstances.**

*RES 8*

#### 3.2.4. Intervention Sustainability

Respondents discussed the wider environment as integral to whole-of-school intervention sustainability. The term ‘wider environment’ describes any setting that is outside of the school environment, including the home, community, or public policy landscape.

Buy-in from parents and key local and national stakeholders to share knowledge and skills, signpost physical activity opportunities, improve facilities and develop policies in support of a broader physically active culture were suggested ways the wider environment could support change at a whole-school level.

**[…] we believe it’s important that when you start something in a school or, equally, when you start something in a community, there is a shared willingness to support that and pathways that either lead from or into either a community or a school opportunity […]**

*RES 7*

**And then there was that buy-in from the parents, which then just creates some- because a lot of this, as well, is about the momentum you can create at home.**

*RES 19*

#### 3.2.5. Addressing Inequalities

When asked about whether a whole-of-school approach could be an opportunity to address inequalities, stakeholders thought that if an approach was framed appropriately, it could address physical activity inequalities by creating an inclusive environment in which individuals feel comfortable.

**I guess it’s taking a proportionate universal approach to interventions that will help reduce those barriers. Doing them for everybody, so that it’s not stigmatising.**

*RES 15*

Several respondents felt that it was crucial to still deliver individual-level interventions to tackle barriers or inequalities, and that these were more likely to be successful because of the culture change induced by the whole-school approach.

**So these individual-level interventions could be run alongside and within the whole-school approach, because the whole-school approach creates the environment for them to work.**

*RES 14*

### 3.3. RQ3: Research Needed to Support Change

Respondents from local and national-level organisations all reported using research to identify and address areas of need, as well as to justify funding applications.

**To justify why you are working in certain places and why you’re trying certain interventions, we will use data and research that has pointed us in that direction.**

*RES 2*

Respondents from national organisations also described repackaging robust research into practical recommendations to empower community-level change and demonstrate the power of different interventions.

**Translating [robust trials] into real clear practice, for schools and even community clubs or whatever, that’s kind of what we try to do to a certain extent, in terms of the insight research that we provide.**

*RES 6*

Respondents from local-level organisations such as local authority councils and regional sports partnerships described how evidence of programme efficacy in their local area was particularly valuable when making decisions about implementing or promoting programmes to schools that they work with.

**The more local a piece of research is and the more relevant it is, the more beneficial I think it is. I think, sometimes, national studies are a bit of a, ‘So what?’**

*RES 2*

Stakeholders implied that such local insight can often take the form of informal communication between schools, rendering such evidence to be anecdotal at times. Using such anecdotal evidence of impact to inform intervention design or provide insight to relevant learnings was commonly stated by stakeholders as being useful because it was from a trusted source. However, respondents identified a need for greater commitment to prioritising robust research alongside such word-of-mouth recommendations, which was also acknowledged.

**Only at such point, where we have all of that information that stands up to academic rigour, could we even begin to understand the societal impact we’re having […]**

*RES 10*

#### Research Needs

Four clear themes emerged in participant responses on this topic. Firstly, several stakeholders described a need for clearer evidence linking physical activity with educational attainment and outcomes specifically scrutinised by Ofsted. Respondents felt that this evidence would be useful for persuading schools to prioritise PA.

**I need to present data that shows that engagement has produced that educational outcome that has improved their chances of educational success or social mobility […].**

*RES 10*

Secondly, an urgent need was expressed for research that describes and evaluates the implementation of whole-school approaches. However, it was recognised that applying the findings from such evaluations to different school settings presents challenges due to the multifaceted nature of a whole-school approach and the uniqueness of each school. Case studies were highlighted as particularly useful to schools considering adoption.

**[…] case studies have always helped me, from a professional point of view, understanding concepts, because if someone gives me a case study of how it’s used in the real world, I can instantly picture in my head […]**

*RES 16*

Third, stakeholders felt that schools and sport sector organisations would benefit from clearer guidance on how to access and use research to design or improve interventions.

**[…] how can we be consistent with our measurement of these interventions and how can we pull it across multiple interventions? Almost that consistency, consistent research tools and frameworks.**

*RES 8*

Fourth, there was a clear appetite for increased collaboration between research institutions, local government, and the sport sector with the aim of improving programme evaluation, informing evidence-based intervention design, and influencing policy.

**[…] it’s always been an aspiration of mine that we have a more formal relationship with universities and research departments, so that when we’re designing something, we’re designing research at the outset, research runs through the programme and then there is a formal evaluation at the end of it.**

*RES 7*

## 4. Discussion

Data in this paper indicate a broad agreement with the existing literature and within the physical activity community that current approaches to increasing physical activity among children and young people have had limited impact. Respondents perceived that intervention design factors, school priorities, ethos and infrastructural barriers were common limiting factors for individual-level intervention impact. Likewise, evaluations of other trials have suggested that implementing their respective programmes in more supportive school environments may lead to increased efficacy [23,24]. Stakeholders felt that another major contributing factor to the failure of intervention-induced behaviour change and its sustainability within schools is the lack of support outside of school, such as poor local community links and a lack of parental engagement. Research [23,25,26,27,28] has previously identified that within-school programmes alone are insufficient to have significant impact on physical activity behaviour, and suggest that wider family, community, and media influences have an important role to play that complement school-based interventions. These concepts are not new and align very closely with the social ecological model of behaviour change, which has been used widely in public health research and practice and which emphasizes the importance of interrelationships between individuals and the social, physical and policy environments they operate in for influencing their behaviour [29]. It seems an error often made is that interventions are being used predominantly to address change at the level corresponding with the individual, as opposed to approaching individual-level change by considering the systems they sit within.

In this study, there was agreement between stakeholders that the adoption of whole-of-school approaches may be a helpful strategy to overcome some of the limitations inherent in individual-level interventions. However, whole-of-school approaches are complex to implement and would involve significant changes, at multiple levels within a school, which may impede adoption in some settings. The World Health Organisation’s (WHO) Health Promoting School (HPS) framework [30] stipulates three fundamental change domains to influence overall health and educational outcomes: formal health curriculum, the ethos and environment of the school, and engagement with families or communities or both. To date, whole-of-school approaches have been used to tackle a wide range of health behaviours [31] but there is no evidence of their use or impact on physical activity in the UK. Physical activity-specific whole-of-school approaches in the UK are, at the time of writing, currently being developed or ongoing, with the results yet to be published [32,33,34]. An example of a whole-of-school approach to physical activity is the ‘Active School Flag’, which has been developed in the Republic of Ireland. This is a programme that comes with a self-evaluation pack, a menu of resources and criteria that must be met by participating schools, and a three-year lifespan which can be renewed with subsequent re-application [35]. An evaluation of these types of programmes in the UK is needed.

In 2019, to address this gap in UK schools, an experience-driven creative consultation between UK academics and public sector stakeholders led to the creation of the Creating Active Schools (CAS) framework [32]. The CAS framework, like the Active School Flag award in Ireland, reinforces the need for change at the individual, school environment, and wider community level, as outlined in the WHO HPS framework [30,31] and underpins some of the current UK pilot schemes for whole-0f-school physical activity interventions. The CAS framework identifies the multiple components needed to establish schools as a complex adaptive sub-system which, in turn, will facilitate whole-school physical activity implementation [32]. It specifies two separate domains—within-school factors, and factors within the wider system beyond an individual school. The cornerstone of the CAS framework is school ethos, sometimes referred to as ‘school culture’. Culture change within schools was discussed extensively by our respondents and, as in the CAS and HPS frameworks, they framed it as both a crucial component to any whole-school approach and as the objective of the approach itself. Their recommendations for ways culture change can be achieved are detailed in Table S2. Commensurate with this, in a recent qualitative study exploring experiences of adopting and implementing a whole-school physical activity programme (‘Transform-Us’) in Australia [36], the results showed that the school culture and values for physical activity among schools taking part positively impacted on intervention implementation by role-modelling and endorsing active behaviour as well as encouraging staff participation in the project. Furthermore, recent qualitative research with schools who had been implementing the Active School Flag in the Republic of Ireland recounts that positive and lasting changes to physical activity provision and participation brought about by participation in the Active School Flag programme were underpinned by changes in the culture around physical activity in those schools [37]. Collectively, these studies and the data presented here highlight a need for a collaborative approach within schools to create a positive culture towards physical activity.

Based on their experience, the stakeholders in this study recommended additional ideas to incorporate into the design of a whole-of-school approach to increasing physical activity that they felt would maximise its efficacy (Table S1). The broader school-level suggestions were to create something that is driven by school needs, strategy and environment that can embed physical activity within the curriculum and create an infrastructure to allow easy integration into the wider community—ideas that align well with the CAS and WHO HPS framework domains. These ideas also align with other whole-school or multicomponent intervention literature, suggesting interventions with such design elements are likely to be successful [31,38,39,40,41,42,43].

Building on the CAS framework recommendations for whole-of-school approach components, our research highlighted key adoption considerations, notably that school leadership must be convinced of the value of the approach for their school and students to consider such a comprehensive change of practice. After evidence of impact, which is still lacking for whole-of-school approaches in UK schools, of primary concern to school leadership is what schools are evaluated against by Ofsted. Thus, to increase adoption, the recommendation of several stakeholders was to employ changes in education assessment policy which prioritise pupil wellbeing and support physical activity beyond P.E. and across the curriculum, thereby promoting classroom initiatives to increase physical activity—a suggestion others have made before [25].

In terms of implementation considerations, stakeholders felt that any whole-of-school approach must work around school-specific challenges including, but not limited to, resource issues, staff capacity and confidence to adapt lessons. These suggestions are consistent with existing qualitative research [37,44,45] and systematic review evidence [42,43] that identify staff capacity, administrative burden and resource issues as key barriers to the implementation and sustainability of a whole-of-school approach to changing behaviour. Additionally, highlighted by Mc Mullen et al. in their research exploring the implementation of the Active School Flag, is the responsibility for change within the school and who shoulders it. Reliance on fewer individuals was identified as a threat to sustainability [37]—a warning also flagged by participants in our study. Bagnall et al. and Ponsford et al. agree that consultation with schools in intervention design is therefore vital to addressing these barriers, creating a flexible and sustainable offer for schools that meets the needs of their community and is deliverable by their staff without reliance on individual champions.

The second domain within the CAS framework is the wider environment, beyond the school gates. In accordance with other research [36,42,43] and existing whole-of-school approach designs [33,34] our respondents discussed factors in this domain as pertaining to the sustainability of interventions and their possible impacts, identifying parental engagement and the creation of pathways between schools and their local communities (that reinforce physical activity messages and create opportunities) as vital to the success of any intervention, including whole-of-school approaches.

### 4.1. Research Recommendations

The views expressed in this paper highlight that fact that stakeholders felt that evidence for the promotion of whole-system approaches to children’s physical activity in the UK was currently unavailable and the evidence that is available does not provide the information that schools need. Stakeholders expressed a desire for information on impacts of physical activity participation on educational attainment and evidence of whole-of-school approach implementation and impact in the form of case studies alongside broader randomised controlled trials. The lack of evidence linking health-promoting school programmes to educational attainment has been highlighted by others [44] and this reinforces the need for related outcomes to be included in future empirical studies in this area. Moreover, as case studies are an increasingly important part of the overall body of evidence, but at the lower end of the hierarchy of evidence [46] and unable to provide generalisable evidence on their own, it should be possible to produce case study-like reports from within larger, more robust designs via more creative dissemination strategies.

### 4.2. Strengths and Limitations

The major strength of this study is the provision of in-depth information from a range of key stakeholders. This is the only study the authors are aware of that considers a range of expert opinions on the specifics of operationalising a whole-school approach to physical activity in the UK. Other articles have considered the components of such approaches (as in the CAS) but have not considered or articulated the potential considerations for adoption and implementation as we have. We have also reported our findings in accordance with COREQ guidance to demonstrate how the research was conducted and our approaches to maximising rigour.

This study is, however, limited by the relatively small sample, which was recruited from the UK, meaning that we are unable to generalise to other settings. It is important to highlight that this research took place between October 2020 and January 2021, when the UK was under national COVID-19 restrictions that limited what schools could offer by way of physical activity opportunities. It is possible that participant views could have been influenced by the restrictions to normal operations and the resultant impact on physical activity participation in schools at the time of interview. It is also important to recognise that we have used a thematic analysis for our data. This method was selected due to the exploratory nature of our analysis and our desire to guide future research and policy in this area. However, we accept that there are many different methods of analysing qualitative data and that other researchers may have opted for a different methodological approach. Finally, participants were aware of the purpose of the study at the point of recruitment, and it is therefore possible that we recruited stakeholders with broadly consistent views with a lack of conflicting ideas and perspectives.

## 5. Conclusions

Current school-based physical activity interventions use an individual-level approach which research has shown is inconsistently and minimally effective at best, and ineffectual in most cases. Critically, many of these interventions have design flaws, such as failure to consult the end user, and are delivered in unsupportive physical, cultural and social environments. Consensus between physical activity stakeholders interviewed for this study points towards the use of a whole-of-school approach to increase physical activity, with careful consideration given to how school culture can and needs to be shifted, working with schools to tailor the approach and circumnavigate staff capacity issues, and building relationships within and outside of the school gates to enhance sustainability. Furthermore, this research recommends a shift in research foci towards building an evidence base around educational outcomes (to support adoption of future whole-school approaches) and whole-school implementation insights (to maximise the operational viability of subsequently created whole-of-school programmes).

## Figures and Tables

**Table 1:** Participant characteristics.

Organisation Type	Role Type	Gender (N)	
		F	M
Local authority	Physical Activity Lead	2	
	Public Health Practitioner		1
	School Partnership Lead	2	
Charity	Project Manager		1
	Physical Activity Specialist		1
	Head of Sport	1	
	Education/Insight Officer	1	1
Government agency	Health and Wellbeing Lead		1
	Education Lead	1	1
Local sports partnership	Children and Young People Manager	1	
	Senior Manager		1
	Sports Development Manager		1
Local delivery pilot	Programme officer		1
Public body	Children and Young People Manager	1	
School	Senior Staff	1	
**Total**		**10**	**9**

## Data Availability

Data generated in this study will be made available in the University of Bristol repository (https://data.bris.ac.uk/data/) once the final report has been published, which we envisage will be by the end of 2021.

## Supplementary Materials

The following are available online at https://www.mdpi.com/article/10.3390/ijerph18147628/s1, Table S1: design and implementation ideas for a successful whole-school approach, Table S2: considerations for successful school-level culture change to support increased physical activity.

## Short Biography of Authors

**Byron Tibbitts** is a project manager who conducts research on physical activity in children and adolescents.

**Kathryn Willis** is a research associate who led the process evaluation on the PLAN-A RCT and the PLAN-A feasibility trial.

**Tom Reid** is a fieldworker who has contributed to research on physical activity in young children and adolescents.

**Russell Jago** was the Principal Investigator of the work. He conducts research in relation to children’s physical activity and many of his individual-level interventions have not been successful when evaluated in robust trials.

**Simon J. Sebire** was a co-investigator and, like R.J., has found limited success of individual-level school-based physical activity interventions. He is also Chief Executive of a charity which is exploring whole-school approaches to physical activity.

**Rona Campbell** was a co-investigator of the work and leads programmes of research concerned with multiple risk behaviours in adolescence and health promotion in schools. She is also involved in Cochrane Reviews of the WHO Health Promoting School framework for improving the health and wellbeing of students and staff.

**Ruth R. Kipping** was a co-investigator of the work and undertakes research concerned with obesity prevention in children and multiple risk behaviours in adolescence.

**Rebecca Kandiyali** is a health economist specialising in child and maternal health. She has a keen interest in the evidential needs of decision makers and is an NIHR impact champion.

## References

[1] Strong W.B., Malina R.M., Blimkie C.J., Daniels S.R., Dishman R.K., Gutin B., Hergenroeder A.C., Must A., Nixon P.A., Pivarnik J.M. (2005). Evidence based physical activity for school-age youth. J. Pediatr..

[2] Poitras V.J., Gray C.E., Borghese M.M., Carson V., Chaput J.P., Janssen I., Katzmarzyk P.T., Pate R.R., Gorber S.C., Kho M.E. (2016). Systematic review of the relationships between objectively measured physical activity and health indicators in school-aged children and youth. Appl. Physiol. Nutr. Metab..

[3] Janssen I., Leblanc A.G. (2010). Systematic review of the health benefits of physical activity and fitness in school-aged children and youth. Int. J. Behav. Nutr. Phys. Act..

[4] Tarp J., Child A., White T., Westgate K., Bugge A., Grontved A., Wedderkopp N., Andersen L.B., Cardon G., Davey R. (2018). Physical activity intensity, bout-duration, and cardiometabolic risk markers in children and adolescents. Int. J. Obes..

[5] Biddle S.J.H., Ciaccioni S., Thomas G., Vergeer I. (2019). Physical activity and mental health in children and adolescents: An updated review of reviews and an analysis of causality. Psychol. Sport Exerc..

[6] Jago R., Salway R., Emm-Collison L., Sebire S.J., Thompson J.L., Lawlor D.A. (2020). Association of BMI category with change in children's physical activity between ages 6 and 11 years: A longitudinal study. Int. J. Obes..

[7] Guthold R., Stevens G.A., Riley L.M., Bull F.C. (2020). Global trends in insufficient physical activity among adolescents: A pooled analysis of 298 population-based surveys with 1.6 million participants. Lancet Child Adolesc. Health.

[8] Corder K., Schiff A., Kesten J.M., van Sluijs E.M.F. (2015). Development of a universal approach to increase physical activity among adolescents: The GoActive intervention. BMJ. Open.

[9] Demetriou Y., Honer O. (2012). Physical activity interventions in the school setting: A systematic review. Psychol. Sport Exerc..

[10] Love R., Adams J., van Sluijs E.M.F. (2019). Are school-based physical activity interventions effective and equitable? A meta-analysis of cluster randomized controlled trials with accelerometer-assessed activity. Obes. Rev..

[11] Rose G. (2001). Sick individuals and sick populations. Int. J. Epidemiol..

[12] Pang B., Kubacki K., Rundle-Thiele S. (2017). Promoting active travel to school: A systematic review (2010–2016). BMC Public Health.

[13] Chillón P., Gálvez-Fernández P., Huertas-Delgado F.J., Herrador-Colmenero M., Barranco-Ruiz Y., Villa-González E., Aranda-Balboa M.J., Saucedo-Araujo R.G., Campos-Garzón P., Molina-Soberanes D. (2021). A School-Based Randomized Controlled Trial to Promote Cycling to School in Adolescents: The PACO Study. Int. J. Environ. Res. Public Health.

[14] Webster C.A., Rink J.E., Carson R.L., Moon J., Gaudreault K. (2020). The Comprehensive School Physical Activity Program Model: A Proposed Illustrative Supplement to Help Move the Needle on Youth Physical Activity. Kinesiol. Rev..

[15] Tong A., Sainsbury P., Craig J. (2007). Consolidated criteria for reporting qualitative research (COREQ): A 32-item checklist for interviews and focus groups. Int. J. Qual. Health Care J. Int. Soc. Qual. Health Care.

[16] Willis K., Tibbitts B., Sebire S.J., Reid T., MacNeill S.J., Sanderson E., Hollingworth W., Kandiyali R., Campbell R., Kipping R.R. (2019). Protocol for a cluster randomised controlled trial of a Peer-Led physical Activity iNtervention for Adolescent girls (PLAN-A). BMC Public Health.

[17] Jago R.T., Tibbitts B., Willis K., Sanderson E., Kandiyali R., Reid T., Kipping R.R., Campbell R., MacNeill S.J., Hollingworth W. (2021). Effectiveness and cost-effectiveness of the PLAN-A intervention, a peer led physical activity program for adolescent girls: Results of a cluster randomised controlled trial. Int. J. Behav. Nutr. Phys. Act. Press.

[18] Patton M.Q. (2002). Qualitaive Research and Evaulative Methods.

[19] Malterud K., Siersma V.D., Guassora A.D. (2016). Sample Size in Qualitative Interview Studies: Guided by Information Power. Qual. Health Res..

[20] Thomas D.R. (2006). A general inductive approach for analyzing qualitative evaluation data. Am. J. Eval..

[21] Saunders B., Sim J., Kingstone T., Baker S., Waterfield J., Bartlam B., Burroughs H., Jinks C. (2018). Saturation in qualitative research: Exploring its conceptualization and operationalization. Qual. Quant..

[22] Cote L., Turgeon J. (2005). Appraising qualitative research articles in medicine and medical education. Med. Teach..

[23] Fairclough S.J., Hackett A.F., Davies I.G., Gobbi R., Mackintosh K.A., Warburton G.L., Stratton G., van Sluijs E.M.F., Boddy L.M. (2013). Promoting healthy weight in primary school children through physical activity and nutrition education: A pragmatic evaluation of the CHANGE! randomised intervention study. BMC Public Health.

[24] de Meij J.S., van der Wal M.F., van Mechelen W., Chinapaw M.J. (2013). A mixed methods process evaluation of the implementation of JUMP-in, a multilevel school-based intervention aimed at physical activity promotion. Health Promot. Pract..

[25] Tymms P.B. (2016). Clustered randomised controlled trial of two education interventions designed to increase physical activity and well-being of secondary school students: The MOVE Project. BMJ Open.

[26] Wake M. (2018). The failure of anti-obesity programmes in schools. BMJ.

[27] Verloigne M., Bere E., Van Lippevelde W., Maes L., Lien N., Vik F.N., Brug J., Cardon G., De Bourdeaudhuij I. (2012). The effect of the UP4FUN pilot intervention on objectively measured sedentary time and physical activity in 10–12 year old children in Belgium: The ENERGY-project. BMC Public Health.

[28] Adab P., Pallan M.J., Lancashire E.R., Hemming K., Frew E., Barrett T., Bhopal R., Cade J.E., Canaway A., Clarke J.L. (2018). Effectiveness of a childhood obesity prevention programme delivered through schools, targeting 6 and 7 year olds: Cluster randomised controlled trial (WAVES study). BMJ.

[29] Stokols D. (1996). Translating social ecological theory into guidelines for community health promotion. Am. J. Health Promot.

[30] WHO Health–Promoting Schools: A Healthy Setting for Living, Learning and Working. www.who.int/school_youth_health/media/en/92.pdf.

[31] Langford R., Bonell C.P., Jones H.E., Pouliou T., Murphy S.M., Waters E., Komro K.A., Gibbs L.F., Magnus D., Campbell R. (2014). The WHO Health Promoting School framework for improving the health and well-being of students and their academic achievement. Cochrane Database Syst. Rev..

[32] Daly-Smith A., Quarmby T., Archbold V.S.J., Corrigan N., Wilson D., Resaland G.K., Bartholomew J.B., Singh A., Tjomsland H.E., Sherar L.B. (2020). Using a multi-stakeholder experience-based design process to co-develop the Creating Active Schools Framework. Int. J. Behav. Nutr. Phys. Act..

[33] Wilson D.D.-S., A. Holmes I. (2020). Sport England Local Delivery Pilot: Join Us: Move. Play (JU:MP). https://ec.europa.eu/health/sites/default/files/state/docs/2020_healthatglance_rep_en.pdf.

[34] The-Health-Improvement-Commission Guernsey Child Physical Activity Surveillance Study. https://healthimprovement.gg/news/article/Guernsey-Child-Physical-Activity-Surveillance-Study.

[35] Flag A.S. https://activeschoolflag.ie/.

[36] Cassar S., Salmon J., Timperio A., Koch S., Koorts H. (2020). A qualitative study of school leader experiences adopting and implementing a whole of school physical activity and sedentary behaviour programme: Transform-Us!. Health Educ..

[37] McMullen J.M., Ní Chróinín D., Iannucci C. (2020). What happened next? Exploring the sustainability of a whole-of-school physical activity initiative. Int. J. Health Promot. Educ..

[38] van de Kop J.H., van Kernebeek W.G., Otten R.H.J., Toussaint H.M., Verhoeff A.P. (2019). School-Based Physical Activity Interventions in Prevocational Adolescents: A Systematic Review and Meta-Analyses. J. Adolesc. Health.

[39] Cohen K.E., Morgan P.J., Plotnikoff R.C., Callister R., Lubans D.R. (2015). Physical activity and skills intervention: SCORES cluster randomized controlled trial. Med. Sci. Sports Exerc..

[40] Grydeland M., Bergh I.H., Bjelland M., Lien N., Andersen L.F., Ommundsen Y., Klepp K.-I., Anderssen S.A. (2013). Intervention effects on physical activity: The HEIA study—A cluster randomized controlled trial. Int. J. Behav. Nutr. Phys. Act..

[41] Pearson M., Chilton R., Wyatt K., Abraham C., Ford T., Woods H.B., Anderson R. (2015). Implementing health promotion programmes in schools: A realist systematic review of research and experience in the United Kingdom. Implement. Sci..

[42] Hunt P., Barrios L., Telljohann S.K., Mazyck D. (2015). A Whole School Approach: Collaborative Development of School Health Policies, Processes, and Practices. J. Sch. Health.

[43] Bagnall A.-M., Radley D., Jones R., Gately P., Nobles J., Van Dijk M., Blackshaw J., Montel S., Sahota P. (2019). Whole systems approaches to obesity and other complex public health challenges: A systematic review. BMC Public Health.

[44] Ponsford R., Meiksin R., Bragg S., Crichton J., Emmerson L., Tancred T., Tilouche N., Morgan G., Gee P., Young H. (2021). Co-production of two whole-school sexual health interventions for English secondary schools: Positive choices and project respect. Pilot Feasibility Stud..

[45] Elfrink T.R., Goldberg J.M., Schreurs K.M.G., Bohlmeijer E.T., Clarke A.M. (2017). Positive educative programme. Health Educ..

[46] Murad M.H., Asi N., Alsawas M., Alahdab F. (2016). New evidence pyramid. Evid. Based Med..

