# Supplementary material for: Considerations for Individual-Level Versus Whole-School Physical Activity Interventions: Stakeholder Perspectives
Source: Int J Environ Res Public Health. 2021 Jul 18;18(14):7628. doi: 10.3390/ijerph18147628 (PMC8304121; doi:10.3390/ijerph18147628)
Supplement: Supplementary file 1 [file ijerph-18-07628-s001.zip › Table S1_Design ideas_final.pdf]

Table S1. Design and implementation ideas for a successful whole-school approach

**Changes to curriculum and school strategy**	**Supporting quotes**
Increase physical activity within the curriculum to help take the pressure off the pupils to be active in their free time. For example, building physical activity-related activities in to subjects outside of PE or making changes to the classroom set up, such as removing chairs, to promote physical activity.	*We have spoken to secondary schools about increasing physical activity within curriculum subjects. So if the onus isn't necessarily on young people to use their, inverted commas, "free time" to be active, but that, actually, they can think about ways that their school day can, just by design, be more active*. **RES 1**
Implement a school-driven model of how to approach sport and physical activity. This should adapt to the school needs and environment and embed ideas throughout the school.	*It doesn't have to work exactly like what it's laid out on a piece of paper. It can be adapted based on the makeup of the school, the size of the school, whether it's got access to green space, whether it's a very urbanised school with hardly any facilities, that kind of stuff.* **RES 16**
Evaluate how learning is organised and what facilities are available. Invest in good quality, continuous training to empower staff to implement strategies themselves.	*It is changing maybe the pedagogy, in terms of it might be changing the curriculum offer. It might be changing how you organise learning and maybe changing the facilities […]***RES 8**
Create supportive infrastructure within schools that allows the approach to be integrated into the community and family.	*So the interventions we're developing have also got to integrate into the community and into the family.* **RES 7**
Start with extra-curricular changes and/or areas that are easy and have the potential to lead to a quick wins.	*Schools quite often will start with the areas they can influence [boys] in more quickly, […] to have those quick wins and that direct engagement, but that ultimately, sometimes, does lead to a longer-term process where the offer of extra-curricular then does influence the curriculum.* **RES 8**
**Rebranding physical activity and Physical Education**	
Normalise physical activity/sport by using more relatable messages and language. For example, encouraging activity through socialising.	*For us, it's championing what else you can get out of being physically active. Is it about spending time with mum, for example? Is it about building skills for your CV in leadership and things like that? There are so many other benefits, meeting new people, socialising.* **RES 6**
Ensure that pupils understand the importance of physical activity and how it can be woven into their everyday lives. For example, provide a wide range of physical activity experiences.	*It's about embedding it within the everyday, rather than as an additional piece of work. It's utilising the everydayness of physical activity.* **RES 18**
Rebrand P.E. to focus on health and wellbeing and build physical activity around having fun.	*They [school piloting a whole-school approach] […] completely rebrand[ed] their entire PE department to healthy active lifestyles. It was very much about health and wellbeing rather than PE. They just found the word PE was limiting them. […]***RES 12**
Use sport as a tool for engagement in other programmes, e.g. using sport-related examples in numeracy or literacy programmes.	*[…] find a way to integrate what you're trying to teach into a physical activity session. And then, without them realising it, they're doing a load of maths and running around and having a load of fun.* ***RES 10***

Increase provision for the whole spectrum of physical activity ability.	*Those who like it [sport] but maybe don't have the ability often miss out [on provision]. That's an area where, if you've got a kid that's going to be a fourth team player, that's great. You can have a great life being a fourth team player. Let's not shut down all those opportunities*. **RES 2**
**Use of role models**	
Use celebrities and relatable figures to endorse physical activity /sport to make it 'cool'.	*Making sport cool is important, and their [This Girl Can] campaign materials are really cool. I think that makes a difference. […] celebrity endorsement…Young girls who are at a stage when they're very influenced by celebrities […]. Seeing positive role models of women that they look up to and admire, making sport cool.* **RES 15**
Non-PE staff should act as role models for being active, and deliver clubs. They are particularly relatable for those that do not enjoy being active and can act as an agent to embed activity throughout the curriculum and school.	*Schools have sent non-PE staff onto courses so they can deliver active lessons. There was one example of a maths teacher taking their class to the school gym and then measuring the angles of the basketball court. Just things like that to make being active a central pillar of the school*. **RES 12**
Take advantage of the mother-daughter relationship - mothers can act as role models to encourage female participation and help normalise physical activity among family and community.	*Mum and daughter relationships are becoming stronger…and girls are really wanting to have that one-to-one time with mum. So, could mum be a key influencer in terms of getting girls far more active?* **RES 6**
**Stakeholder consultation**	
Involve pupils in shaping the whole school approach, giving them autonomy and helping designers understand pupils wants and needs.	*[…] co-creation with young people and the intended recipient is increasingly important.* **RES 8**
Consult with head teachers, SLT and other teachers in the design process to increase school engagement and ownership. This is key for creating a high sense of value for the project and successful spearheading of the approach.	*The whole ethos of our whole pilot is to co-design with people rather than for people, because we'd like to think that if people feel like they have a value in something then they'll take ownership of that.* **RES 16**
Involve parents to utilise their inside knowledge of their children and desire to improve their children's quality of life. For example, to understand how to support adolescent mental health or how best to encourage a healthy lifestyle.	*Parents would be an interesting group to talk to about supporting their young person's mental health throughout childhood and adolescence, and the importance of keeping healthy routines*. **RES 1**
Consult local health networks, this will also help gain access to other important stakeholders to engage with.	*Through that health and wellbeing network there would be others that you would look to engage with as well, because they have huge outreach into networks like school nurses*. **RES 4**
Engaging with national organisations in the physical activity sector can help improve intervention design because of their vast experience in implementing physical activity programmes.	*There are some national bodies now, like the Youth Sport Trust, that have a long, long history now of developing projects and ideas around this kind of work, which might help in terms of sharpening up an idea and a project*. **RES 9**
