# Supplementary material for: Considerations for Individual-Level Versus Whole-School Physical Activity Interventions: Stakeholder Perspectives
Source: Int J Environ Res Public Health. 2021 Jul 18;18(14):7628. doi: 10.3390/ijerph18147628 (PMC8304121; doi:10.3390/ijerph18147628)
Supplement: Supplementary file 1 [file ijerph-18-07628-s001.zip › Table S2_ Culture change_final.pdf]

Table S2. Considerations for successful school-level culture change to support increased physical activity

School level considerations	Supporting quotes
Make physical activity-related objectives part of the whole school improvement plan. This will help physical activity become a higher priority in the school and influence the attitude and values the school community place on it.	*We've found getting a whole school approach, there are things we've found work. Getting physical activity embedded in the whole school improvement plan helps.* **RES 12**<br>*In order that it [physical activity] can feature highly in a School Improvement Plan, it needs to have another objective, or Ofsted […] And that be part of the criteria.* **RES 1**
Ensure that there is ongoing logistical and financial support for the school to help make and maintain positive changes, which will in turn continue to support culture change. For example, use funding to promote curriculum changes.	*Inside curriculum is a great place to start, because there is a bit of statutory funding for it, so it's easy for us to build a delivery force that provides brilliant sessions in school.* **RES 10**<br>*[…] some schools are incredibly good at building in sport and physical activity into the whole school ethos, […] and other schools really need a lot of support and help to try and get that message across much more.* **RES 6**
Offer a wider range of inclusive physical activity opportunities within school to help nurture a culture supportive of physical activity. This could be supported by collaborating with the wider community.	*One other way we can really get girls excited about sport and physical activity is actually to connect up with the local community much more through schools…So, having local sports clubs or community centres engaging with the schools and offering facilities.* **RES 6**<br>*The only thing we feel you want to do is to give kids as wide an experience – as positive and as wide an experience – of as many physical activities as early as possible.* **RES 9**
Ensure messaging about physical activity remains positive and consistent throughout the school, this will reinforce school culture and have more impact on pupil's attitude and values. This could be achieved by including a communications plan.	*I think a lot of it is about we've found consistent messaging across the school makes a lot of difference. Making sure the […] why physical activity is important is not just coming from the PE department.* **RES 12**<br>*[…] engaging heads of years to have certain times where it's their tutor talking to them about physical activity and being healthy, rather than the PE teacher. Again, that's what they expect when they speak to us, but if they're hearing the same message from someone outside of the department, in many ways that probably has more of an impact.* **RES 19**
Make positive changes and support for physical activity for all school stakeholders, not just for the pupils or to PE. This could aid intervention buy-in and support for physical activity throughout the school, leading to positive culture change.	*You will have a number of teachers who just don't like physical activity, or a number of teachers who are overweight or obese, who might feel uncomfortable about physical activity themselves. What is the staff culture in relation to staff wellbeing in that school, more broadly?* **RES 13**
Create an environment that is comfortable for pupils to be active in. For example, having appropriate facilities and skilled staff to lead and act as role models can contribute towards a positive physical activity-based culture.	*[…] the facilities and the resources schools have is a major component of that [whole-school approach], do they have the right kind of sports facilities? […] I've been to a variety of different schools and you can see right the way, across the school, that where the provision is good, and they've got the access and the facilities and they've got well trained staff, the sport and physical activity provision can be excellent.* **RES 6**

	[…] there has been some positive stuff around facilities and environments. It's almost trying to get to that point where those adolescents, particularly girls, are accessing that activity somewhere where they feel it's not school, but it is. **RES 9**
**Staff- and pupil-level considerations**	
Buy-in to the intervention and associated changes is needed at all levels throughout the school, particularly from decision makers (SLT, head teacher, governors), to endorse and sustain culture change throughout the school. This has somewhat of a circular effect, because buy-in is needed for changes to happen, yet changes need to happen to help encourage buy-in.	*[…] in order to truly improve the experience of PE for young people it needs to be this culture change that comes from the top in schools. Senior leadership engagement and endorsement is key […].* **RES 12**<br><br>*[…] if you get the buy-in at the top then the rest, kind of, flows down to the staff.* **RES 16**
Encourage positive staff attitudes, passion and drive for physical activity to embed these values into the school ethos. Again, this is likely to be more effective if it comes from the top. This can be influenced by and influence school priorities and intervention buy-in. It also has the potential to have an impact on pupil attitudes towards physical activity	*[…] you've got a leadership there that value what we [PE department] do. And so that's quite important because the students see that as important.* **RES 19**<br><br>*[…] it's not embedded in the ethos that actually mental wellbeing and physical wellbeing is part and parcel of having a successful school career. I think that's what it really needs to be, and I think if that comes from all corners of the school right from the top down, you know, and you embed that within the school I think you're much more likely to have an engaged school.* **RES 6**
The intervention needs championing within school, without the need to rely on one individual. Champions need to have passion and ability to drive change whilst being relatable to pupils. For example, less 'sporty' teachers to act as positive role-models. Confident intervention leadership will also act as a vehicle for culture change.	*It's got to be the right teacher with the right personality. Ideally…It would be a history teacher or an RE teacher, anyone but your typical sporty woman, I think that really is a put-off for a lot of girls. It's intimidating, they can't… They just don't relate to it in any way.* **RES 3**<br><br>*I think you probably do need one person who is leading the overall approach, but it doesn't mean that person should be doing it all. That person is almost project managing it, but it shouldn't fall on one person […].* **RES 15**
Upskill and improve knowledge of all staff about physical activity to aid their understanding and value for its importance, in turn creating positive staff attitudes and driving school culture change.	*Upskilling staff in physical activity training, not just PE staff, but particularly the PE staff, really helps.* **RES 12**<br><br>*So we've created champions in each setting [school] so that we can target them for further bespoke training. But also, we can start to share ideas, problem solve where physical space or resource can be an issue, but really embed that in a really good solid knowledge and understanding of child development […].* **RES 18**
